# Establishment of a 3D esthetic analysis workflow on 3D virtual patient and preliminary evaluation

**DOI:** 10.1186/s12903-024-04085-0

**Published:** 2024-03-13

**Authors:** Kwantong Cheung, Waisze Cheung, Yunsong Liu, Hongqiang Ye, Longwei Lv, Yongsheng Zhou

**Affiliations:** Department of Prosthodontics, Peking University School and Hospital of Stomatology & National Center for Stomatology & National Clinical Research Center for Oral Disease & National Engineering Research Center of Oral Biomaterials and Digital Medical Devices & Beijing Key Laboratory of Digital Stomatology & NHC Key Laboratory of Digital Stomatology & NMPA Key Laboratory for Dental Materials, No. 22, Zhongguancun South Avenue, Haidian District, Beijing, 100081 China

**Keywords:** 3D esthetic analysis, Digital dentistry, Dental Esthetic, 3D virtual patient, Artificial intelligence

## Abstract

**Background:**

In esthetic dentistry, a thorough esthetic analysis holds significant role in both diagnosing diseases and designing treatment plans. This study established a 3D esthetic analysis workflow based on 3D facial and dental models, and aimed to provide an imperative foundation for the artificial intelligent 3D analysis in future esthetic dentistry.

**Methods:**

The established 3D esthetic analysis workflow includes the following steps: 1) key point detection, 2) coordinate system redetermination and 3) esthetic parameter calculation. The accuracy and reproducibility of this established workflow were evaluated by a self-controlled experiment (*n* = 15) in which 2D esthetic analysis and direct measurement were taken as control. Measurement differences between 3D and 2D analysis were evaluated with paired *t*-tests.

**Results:**

3D esthetic analysis demonstrated high consistency and reliability (0.973 < ICC < 1.000). Compared with 2D measurements, the results from 3D esthetic measurements were closer to direct measurements regarding tooth-related esthetic parameters (*P*<0.05).

**Conclusions:**

The 3D esthetic analysis workflow established for 3D virtual patients demonstrated a high level of consistency and reliability, better than 2D measurements in the precision of tooth-related parameter analysis. These findings indicate a highly promising outlook for achieving an objective, precise, and efficient esthetic analysis in the future, which is expected to result in a more streamlined and user-friendly digital design process.

This study was registered with the Ethics Committee of Peking University School of Stomatology in September 2021 with the registration number PKUSSIRB-202168136.

## Background

A comprehensive esthetic analysis plays an important role in the diagnosis and treatment plan in esthetic dentistry [1, 2]. With the development of digital dentistry, a full digital and intelligent workflow is paramount to bridge the process of analysis, diagnosis, treatment plan and restoration [3]. Smile design usually involves the analysis of facial images and intraoral images to assess parameters such as incisal edge position, width-to-length ratio of upper incisor, gingival margin position, width ratio of upper anterior teeth [4, 5], etc. These parameters serve as references to create a photo design of the post-restorative effect, which is known as Digital Smile Design (DSD) [6]. However, this approach basically focuses on the frontal view, lacking depth information, making it insufficient for directly guiding the design of three-dimensional (3D) restorations [7, 8]. Although 3D esthetic analysis is an essential process to link diagnosis and 3D treatment plan, most of the existing 3D analyses were done on photos, namely the projections of 3D dentitions in the frontal view [9–11]. Therefore, it is urgent to establish a workflow of a real 3D esthetic analysis directly on 3D facial and dental models. Furthermore, the application of artificial intelligence (AI) is currently limited to assisting in diagnostics in dentistry, such as detection of periodontal disease [12–14], reconstruction of bone and tooth from CT [15–17] and caries detection [18–20]. These early applications underscore the growing potential of artificial intelligence in the realm of dentistry.

In this context, it is imperative that every esthetic point selected and every formula developed within this workflow can be efficiently identified and executed by computers and artificial intelligence. This would facilitate potential chairside applications in the future. The established 3D esthetic analysis workflow in this study includes the following steps: 1) key point detection, 2) coordinate system redetermination and 3) esthetic parameter calculation. The accuracy and reproducibility of this established workflow were evaluated afterward.

## Study design

Nine esthetic parameters which are tightly related to the treatment plan and restoration design were selected for esthetic analysis. The 9 esthetic parameters were classified into 3 groups: (1) Tooth-related esthetic parameters, including length of upper incisor, width of upper anterior tooth, overbite, and overjet; (2) Dental-facial esthetic parameters, including incisor exposure at rest position, gingival exposure when smiling, distance between incisor midline and facial midline; (3) Facial esthetic parameters, including distance between upper lip and E line, as well as nasolabial angle.

A self-controlled experiment was applied. As for the 4 tooth-related esthetic parameters, the esthetic analyses were done by three methods, i.e. the established 3D esthetic analysis workflow, two-dimensional (2D) esthetic analysis and direct measurement, respectively. And direct measurement was regarded as a gold standard. 3D esthetic analysis workflow or 2D measurements were compared with direct measurement respectively. The other 5 parameters, i.e. dental-facial esthetic parameters and facial esthetic parameters, cannot be measured directly due to the facial movement. Therefore, 3D esthetic analysis workflow of incisor exposure at rest position, gingival exposure when smiling, distance between incisor midline and facial midline, distance between upper lip and E line, as well as nasolabial angle, were compared directly with 2D measurement. Each measurement method was done for three times by three different researchers.

### Object

This study involved 15 healthy individuals (4 men and 11 women) between the age of 25 to 30 who volunteered to participate. Based on the initial results, the sample size was determined using an α of 5% and with a power of 90%. The inclusion criterion was a healthy and intact dentitions. The exclusion criteria were as follows: 1. Tooth or dentition defects in anterior teeth; 2. Tooth crowding or a diastema greater than 1 mm; 3. Prosthetic restorations in anterior teeth, i.e., veneer, crown, bridge; 4. Maxillofacial asymmetry or developmental abnormalities. The written informed consent was acquired from all participants prior to their inclusion in the study.

### Procedure of 3D esthetic analysis workflow

#### Step 1. Acquisition of 3D facial and dental information

**Fig. 1:**
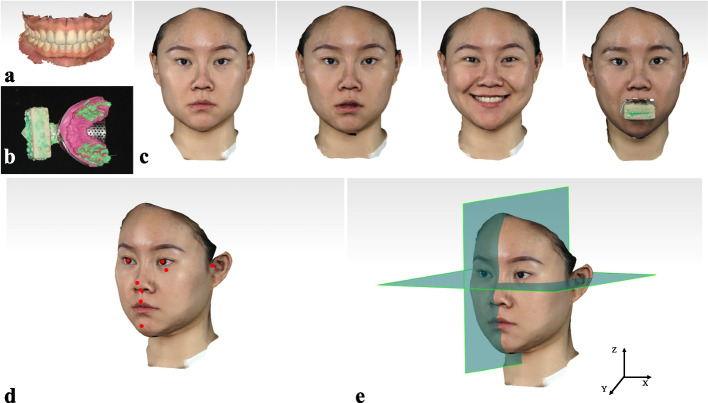
Data acquisition and virtual patient establishment of 3D esthetic analysis workflow. **a** 3D dentitions; **b** The registered-block impression; **c** 3D data acquisition of 3D facial photographs in closed-lip, rest position, wide smile and facial photograph with registered-block impression; **d** Selection of key facial esthetic points; **e** Redetermination of a new coordinate system

An intraoral scan (TRIOS, 3Shape, Denmark) was used to obtain the 3D data of dentitions, i.e. the upper arch and the lower arch in the intercuspal position (Fig. 1a). The oral scan was then exported in Polygon File Format (PLY). To ensure the accuracy of registration between 3D dentitions and 3D facial information, a maxillary impression was made with silicone rubber (Variotime, Kulzer, Germany) and a registered-block was then fixed on the handle of the impression (Fig. 1b). The impression with registered-block was then scanned by an optical scanner (D2000, 3Shape, Denmark). The 3D facial information was obtained by Face Scan (Face Scan, 3D-SHAPE, Germany) in closed-lip, rest position, wide smile and with the registered-block impression (Fig. 1c). Before facial scanning, the left infraorbital point and the superior points of the bilateral external auditory meatus were marked on the volunteers’ faces. The 3D facial models were exported in Wavefront Object format (OBJ) with color.

#### Step 2. Redetermination of coordinate system and establishment of 3D virtual patient

The reverse engineering software (Geomagic Studio, 3D System, USA) was used to import all of the data. A new coordinate system was redetermined on the closed-lip facial scan. Three points, the left infraorbital point and the superior points of the bilateral external auditory meatus, on the closed-lip facial scan determined the XY plane. The Z axis, perpendicular to the XY plane, passed through the midpoint of interpupillary line (Fig. 1e). Finally, all the other facial images were registered with the closed-lip facial image, and the oral scans were registered to the facial image with registered-block impression as described in our previous study [21]. Hence, a 3D dento-facial virtual patient with 3D facial information and 3D dentitions was established in the new coordinate system.

#### Step 3. Selection and recognition of key esthetic points and contours

The key esthetic points and contours were selected according to the minimum needs of the follow-up steps of esthetic parameter calculation. The bilateral pupils, left infraorbital point, the superior points of the bilateral external auditory meatus, nasal tip, nasal columella, nasal base, pogonion, midpoint of upper lip on the closed-lip facial scan and the contour of the upper lip on the closed-lip, rest position and wide smile facial scan, and the contours of each upper anterior tooth and lower incisor were selected as key esthetic points and contours. As mentioned above, the bilateral pupils, left infraorbital point and the superior points of the bilateral external auditory meatus were used to determine the head posture in a new coordinate system. Nasal tip, nasal columella, nasal base, pogonion, midpoint of upper lip and the contour of lips were used for facial esthetic analysis and lip-tooth relation analysis (Fig. 1d). The border line was drawn along the gingival margin on each upper anterior tooth and lower incisor to section each tooth from the dentition model.

#### Step 4. 3D esthetic parameter calculation

Nine esthetic parameters, closely related to the treatment plan and restoration design, were carefully selected as outlined in the study design. The calculation formulas for these 3D esthetic parameters were estabilished as follows.

**Fig. 2:**
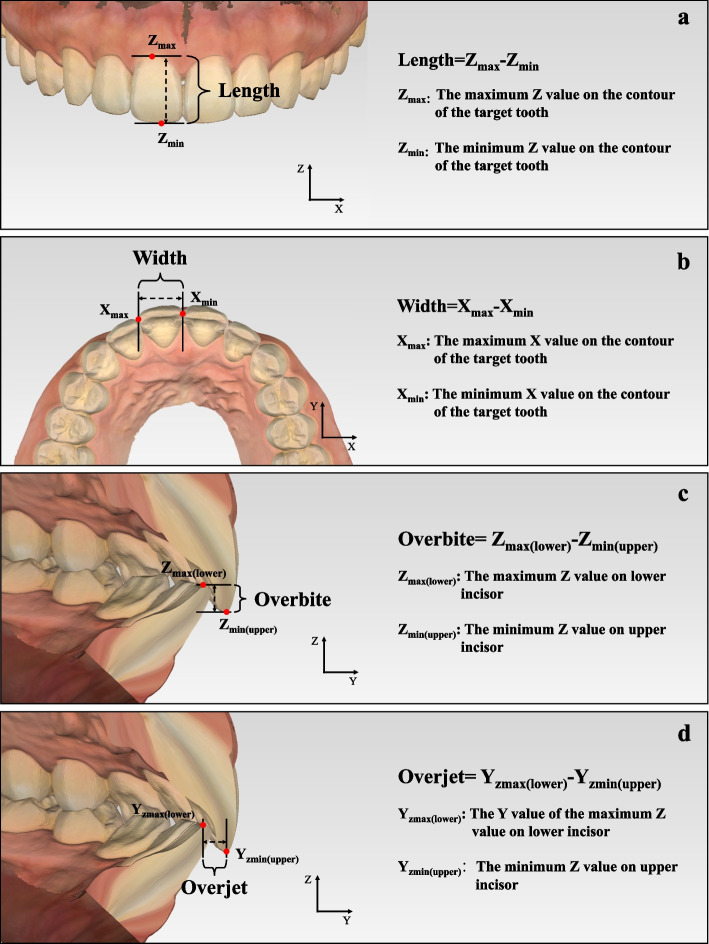
Tooth-related esthetic parameters analysis of 3D esthetic analysis workflow. **a** Length of upper incisor; **b** Width of upper anterior tooth; **c** Overbite; **d** Overjet

**Fig. 3:**
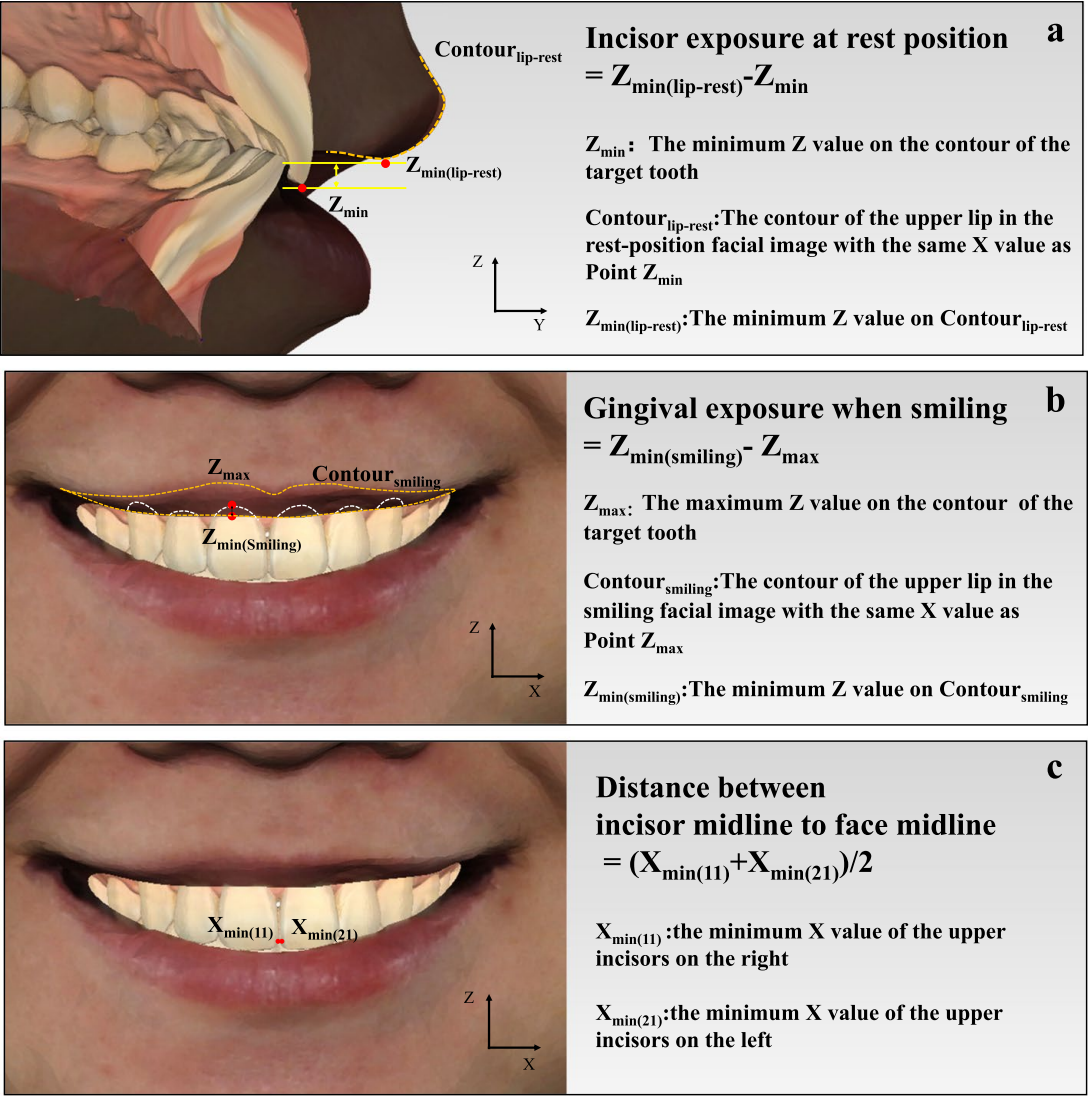
Dental-facial esthetic parameters analysis of 3D esthetic analysis workflow. **a** Incisor exposure at rest position; **b** Gingival exposure when smiling; **c** Distance between incisor midline and facial midline

**Fig. 4:**
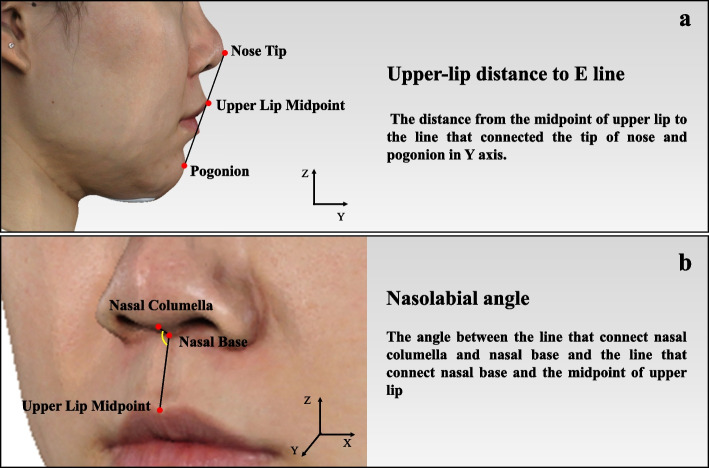
Facial esthetic parameters analysis of 3D esthetic analysis workflow. **a** Distance between upper lip and E line; **b** Nasolabial angle

1. Tooth-related esthetic parameters
  1. **Length of upper incisor**: the difference between the maximum and the minimum *Z* value on the contour of the target tooth (Fig. 2a). The calculation formula is: L = Z_max_-Z_min_.
  2. **Width of upper anterior tooth**: the difference between the maximum and minimum *X* value on the contour of the target tooth (Fig. 2b). The calculation formula is: W = X_max_-X_min_.
  3. **Overbite**: The difference between the minimum *Z* value on upper incisor and the maximum *Z* value on lower incisor (Fig. 2c). The calculation formula is: Overbite = Z_max(lower)_-Z_min(upper)_.
  4. **Overjet**: The difference between the *Y* value of the maximum *Z* value on lower incisor and the minimum *Z* value on upper incisor (Fig. 2d). The calculation formula is: Overjet = Y_Zmax(lower)_-Y_Zmin(upper)_.
2. Dental-facial esthetic parameters
  1. **Incisor exposure at rest position**: The point with the minimum *Z* value on the contour of the target tooth was defined as Point Z_min_. The contour of the upper lip in the rest-position facial image with the same *X* value as Point Z_min_ was selected as the target contour (Contour_lip-rest_). Then incisor display at rest position was determined as the difference between the minimum *Z* value on Contour_lip-rest_ and the *Z* value of Point Z_min._ Therefore, if the incisors could not be exposed at the rest position, the difference would be a negative number (Fig. 3a). The calculation formula is: Incisor exposure at rest position = Z_min(lip-rest)_-Z_min_.
  2. **Gingival exposure when smiling**: The point with the maximum *Z* value on the contour of the target tooth was defined as Point Z_max_. The contour of the upper lip in the smiling facial image with the same *X* value as Point Z_max_ was selected as the target contour (Contour_smiling_). Then gingival exposure when smiling was determined as the difference between the minimum *Z* value on Contour_smiling_ and the *Z* value of Point Z_max._ If the gingiva could not be exposed when smiling, the difference was recorded as zero (Fig. 3b). The calculation formula is: Gingival exposure when smiling = Z_min(smiling)_- Z_max_.
  3. **Distance between incisor midline and facial midline:** the mean value of the minimum *X* value of the two upper incisors (Fig. 3c). The calculation formula is: Distance between incisor midline and facial midline = (X_min(11)_ + X_min(21)_)/2
3. Facial esthetic parameters
  1. **Distance of upper lip and E line**: The distance from the midpoint of upper lip to the line that connected the tip of nose and pogonion in Y axis (Fig. 4a).
  2. **Nasolabial angle**: The angle between the line that connect nasal columella and nasal base and the line that connect nasal base and the midpoint of upper lip (Fig. 4b).

### Procedure of 2D esthetic analysis

#### Step 1. Photograph taking

**Fig. 5:**
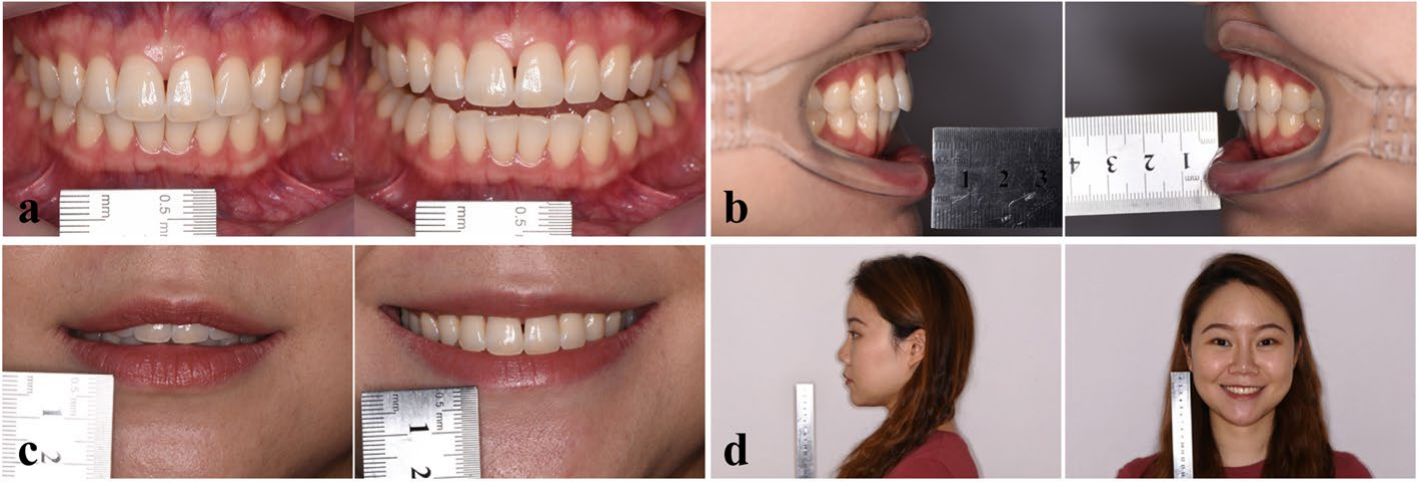
Photograph taking for 2D esthetic analysis. **a **Measurements of lengths of upper incisors, widths of upper anterior teeth and overbite;** b **Measurement of overjet;** c **Measurements of incisor exposure at rest position and gingival exposure when smiling;** d **Measurements of distance between incisor midline and facial midline, distance between upper lip and E line, as well as nasolabial angle

Photos were taken by a Digital Single-Lens Reflex (DSLR) camera (EOS 80D, Canon, Japan) with micro lens (EF 100 mm f/2.8 L IS USM, Canon, Japan) and micro ring flash (MR-14EX II, Canon, Japan) that was mounted on a tripod. The lighting conditions in the room remained consistent during the photo shoot. Photographs of the volunteers were captured while they were seated with their heads held in a naturally upright position. For each volunteer, a total of 8 2D photographs were obtained. These included two intraoral photographs taken with an extractor to open the mouth, two lateral photographs of the dentitions, one photograph illustrating the tooth-lip relationship in the rest position, one photograph showing the tooth-lip relationship when smiling, one frontal portrait captured when smiling, and one lateral profile photograph with the mouth in a naturally closed position. Volunteers were seated upright with a ruler displayed in the camera frame as a reference for measurement (Fig. 5).

#### Step 2. 2D esthetic analysis

Nine esthetic parameters, including 4 tooth-related esthetic parameters (length of upper incisor, width of upper anterior tooth, overbite and overjet), 3 dental-facial esthetic parameters (incisor exposure at rest position, gingival exposure when smiling and distance between incisor midline and facial midline), and 2 facial esthetic parameters (distance between upper lip and E line, as well as nasolabial angle), were measured on photos. The photos were processed in PowerPoint (Office 2016, Microsoft, USA) as mentioned in the previous study of digital smile design [22, 23]. The esthetic parameters were then converted according to the length of the ruler showed in the photos Fig. 5.

### Direct esthetic analysis

#### Step 1. Impression taking

The upper and lower arches were recorded with silicone rubber impressions (Variotime, Kulzer, Germany), and plaster casts (Royal Rock Type 5, Pemaco, US) were subsequently fabricated.

#### Step 2. Direct measurement

**Fig. 6:**
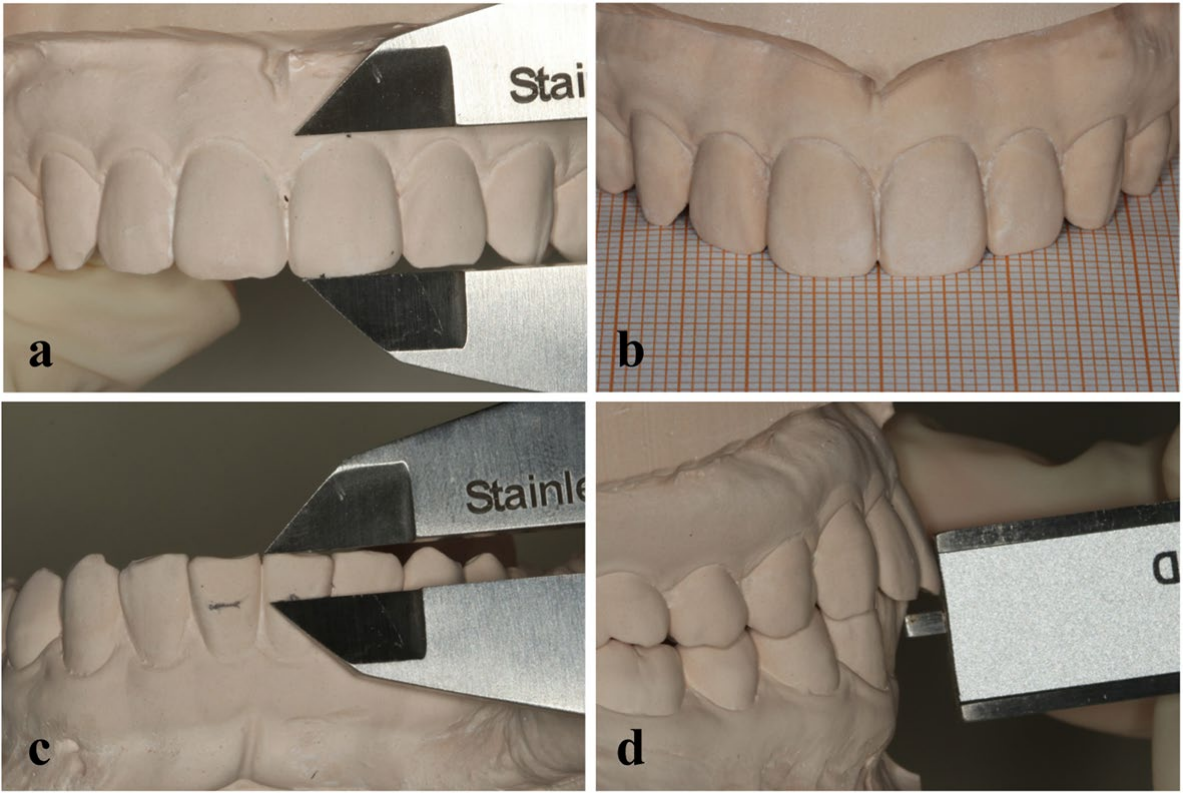
Direct measurement. **a** Lengths of upper incisors; **b** Widths of upper anterior teeth were measured from the frontal view of the plaster model on the millimeter paper by using caliper; **c** Overbite of the upper anterior teeth; **d** Overjet of the upper anterior teeth

Four tooth-related esthetic parameters, including length of upper incisor, overbite and overjet, were measured directly on the plaster model by a caliper. The widths of upper anterior teeth were marked from the frontal view of the plaster model on the millimeter paper and subsequently measured by a caliper (Fig. 6).

## Statistical analysis

Data were analyzed by using SPSS (SPSS Statistics 24; IBM, USA). To evaluate reproducibility, intraclass correlation coefficient (ICC) was used to calculate the repeated measurements of 3D analysis and 2D analysis to evaluate consistency and reliability. To evaluate the accuracy of lengths of upper incisors, widths of upper anterior teeth, overjet and overbite, Shapiro-Wilk test and homogeneity of variance test were applied to evaluate the normality of the difference between 3D esthetic analysis workflow and direct measurement, as well as the difference between 2D measurement and direct measurement. According to the result of Shapiro-Wilk test and Levene's test for normality and homogeneity of variance, respectively, paired *t-*test or Mann-Whitney U test was then used to evaluate the measurement difference about these items of two methods and *P*-values less than 0.05 were considered statistically significant.

## Results

### Consistency and reliability evaluation of three measurement methods

To evaluate consistency and reliability, the ICC of 3D esthetic analysis workflow, 2D esthetic analysis were 0.973 < ICC < 1.000, 0.859 < ICC < 0.992 respectively (Table 1) which indicate that the reproducibility of all methods is high.

**Table 1:** Intraclass correlation coefficient values of 3D esthetic analysis workflow and 2D esthetic analysis methods

Items	3D esthetic analysis workflow	2D esthetic analysis	*n*
Length of upper incisor	0.999	0.963	30
Width of upper anterior tooth	0.999	0.992	90
Overbite	0.998	0.982	30
Overjet	1.000	0.876	30
Incisor exposure at rest position	0.998	0.981	30
Gingival exposure when smiling	0.996	0.984	30
Distance between incisor midline and facial midline	0.987	0.859	15
Distance between upper lip and E line	0.993	0.897	15
Nasolabial angle	0.973	0.916	15

### Accuracy of 3D and 2D esthetic analysis with direct measurement in tooth-related esthetic parameters

To evaluate the accuracy, the difference between 3D esthetic analysis workflow and direct measurement, as well as the difference between 2D measurement and direct measurement were calculated and the two differences were compared with paired *t*-test (Table 2). In the length of upper incisor, the width of upper anterior tooth, overjet and overbite, the difference between 3D esthetic analysis workflow and direct measurement were 0.00 ± 0.28 mm, 0.09 ± 0.31 mm, 0.01 ± 0.02 mm and 0.01 ± 0.02 mm respectively, the difference between 2D analysis and direct measurement were 0.26 ± 0.29 mm, − 0.10 ± 0.29 mm, 0.14 ± 0.33 mm and 0.21 ± 0.45 mm respectively, which means the results of 3D esthetic analysis workflow in tooth-related esthetic parameters were closer to direct measurement and the difference between 3D esthetic analysis workflow and direct measurement found statistically significant with the difference between 2D analysis and direct measurement (*P* < 0.05).

**Table 2:** Comparison between 3D esthetic analysis workflow and 2D esthetic analysis of tooth-related esthetic parameters

Items	Difference between 3D esthetic analysis workflow and direct measurement (mean ± SD, mm)	Difference between 2D esthetic analysis and direct measurement (mean ± SD, mm)	*P*-value*	*n*
Length of upper incisor	0.00 ± 0.28	0.26 ± 0.29	<0.001	30
Width of upper anterior tooth	0.09 ± 0.31	−0.10 ± 0.29	<0.001	90
Overbite	0.01 ± 0.02	0.14 ± 0.33	0.039	30
Overjet	0.01 ± 0.02	0.21 ± 0.45	0.031	30

*Paired *t*-test of the difference between 3D esthetic analysis workflow and direct measurement as well as the difference between 2D esthetic analysis and direct measurement

### The difference between two esthetic analysis of dental-facial and facial esthetic parameters

The mean and SD of dental-facial and facial esthetic parameters for two methods are presented in Table 3. There was no significant difference in the measurement of gingival exposure when smiling, distance between incisor midline and facial midline and distance between upper lip and E line (0.306 < *P* < 0.733). However, there was significant difference in the measurement of the incisor exposure at rest position and nasolabial angle (*P* < 0.05).

**Table 3:** Difference of 3D esthetic analysis workflow and 2D esthetic analysis of dental-facial and facial esthetic parameters

Items	3D esthetic analysis workflow (mean ± SD, mm)	2D esthetic analysis (mean ± SD, mm)	*P*-value*	*n*	Mean of the difference (mean ± SD, mm)
Incisor exposure at rest position	2.99 ± 1.66	2.44 ± 1.26	0.001	30	0.55 ± 0.74
Gingival exposure when smiling	0.60 ± 1.02	0.56 ± 0.97	0.306	30	0.04 ± 0.22
Distance between incisor midline and facial midline	0.14 ± 1.59	0.06 ± 0.94	0.733	15	0.09 ± 1.5
Distance between upper lip and E line	1.22 ± 1.60	1.34 ± 1.43	0.650	15	−0.12 ± 1.00
Nasolabial angle	96.36 ± 8.02	89.86 ± 9.03	0.003	15	6.5 ± 7.07

*Paired *t*-test of the difference between 3D esthetic analysis workflow and 2D esthetic analysis

## Discussion

In order to build a 3D esthetic analysis workflow that could be run easily by computers automatically, key esthetic points and contours were selected and a series of esthetic analysis formulas were established in this study. This study included volunteers with healthy and intact dentitions to verify the accuracy of the esthetic calculation formulas. Patients with esthetic defects in the anterior teeth, such as tooth or dentition defects, and diastema, will be measured and analyzed in our future studies. Meanwhile, the consistency and reliability of this newly established 3D esthetic analysis workflow were evaluated and confirmed. There were some reported 3D measuring methods [24, 25], but the existing methods cannot be used for AI recognition or computer automatic calculation because the key measuring points and the calculation formula were not exactly defined. The current facial key point recognition methods have not yet established a system of key esthetic points that can be applied specifically to dental esthetic restorations [26–29]. Actually, the accuracy of 3D analysis could be affected by the changes of observation angle due to the rotation of facial scan [24, 25, 30]. Therefore, it is essential to maintain the head posture of 3D virtual patient during the whole process of esthetic analysis. To ensure the reproducibility of 3D analysis, the esthetic analysis should be done at a repeatable head posture. Therefore, a new coordinate system based on an adjusted head posture was determined. The Frankfort horizontal plane is an anatomical plane that is defined by the left infraorbital point and the superior points of the bilateral external auditory meatus [31]. This plane is parallel to the horizon, and therefore, the left infraorbital point and the superior points of the bilateral external auditory meatus are used to establish the horizontal plane of the coordinate system. In this new coordinate system, the key esthetic points could be defined exactly. For instance, the length of upper incisor can be measured using the formula L = Z_max_-Z_min_, where the *Z*-value corresponds to the highest and lowest points when viewed from the front perspective.

Although the reproducibility of both 3D esthetic analysis workflow and 2D analysis were satisfactory, the difference between 3D analysis and direct measurements was smaller in tooth-related esthetic parameters compared with 2D analysis. In studies related to anterior dental esthetics, digital smile design method is frequently used for preoperative esthetic analysis and design purposes [7, 9, 11, 23, 32, 33]. The intraoral scanner utilized in this study has been previously demonstrated to possess a high level of accuracy, as evidenced by previous research [34, 35]. In regards to the comparison between intraoral scanning and photograph, previous studies have conducted direct comparisons by comparing screenshots of anterior dental views obtained through intraoral scanning with frontal photographs taken by a DSLR camera. These studies have found no statistically significant differences in the lengths and widths of the maxillary anterior teeth [36]. As in the study done by Ahmed et al., the 3D measurement of mesiodistal widths of upper anterior teeth was done by measuring contact point from the facial side and the result had significant difference with the measurement on the photos [37].

In the process of direct measurement, clear advantages were observed in measuring dental-facial and facial esthetic parameters using the 3D esthetic analysis workflow. The incisor exposure at rest position and gingival exposure when smiling were affected by the muscle tightness of lips which were hard to measure by direct measurement. In addition, keeping the lips in a particular level during direct measurement would cause shaking, thus leading to poor repeatability and affecting accuracy. On the other hand, the facial scan machine only needs 0.8 seconds to acquire 3D facial information which avoided shaking of lips and enhanced patient friendliness [38]. The distance between incisor midline and facial midline, distance between upper lip and E line, as well as nasolabial angle, were also hard to obtain directly due to the short distance and elasticity of soft tissue. However, 3D esthetic analysis workflow and 2D analysis can both overcome these shortcomings. Therefore, a direct comparison was made between them for these esthetic items, and the 3D esthetic analysis workflow still demonstrated a significant advantage. To measure the nasolabial angle, 3D facial scans can capture more facial details, such as the starting point of nasal columella, nasal base and the midpoint of upper lip in three dimensions. This allows for a more accurate measurement of the angle between the lines connecting these points. In contrast, 2D measurement relies on the lateral projection of a photograph, which only shows the apparent angle between these lines on a flat plane and can be easy influenced by the angle of camera and posture. When determining the nasolabial angle from a profile image, the presence of the nostril in the image may obscure the precise location of the measurement point, which can lead to inaccuracies in the obtained results. When measuring incisal exposure at rest position, the 3D esthetic analysis workflow selects an objective value from the 3D model for the incisal point, whereas the 2D measurement subjectively selects a measurement point by the surveyor, which may not represent the actual incisal point and therefore may produce a lower measurement value compared to 3D. Therefore, while both 3D and 2D measurements can overcome the difficulty of directly measurement on face, 3D esthetic analysis workflow may have an advantage in analyzing nasal and dental-facial information.

Through an extensive database and complex neural network computations, tasks that were once thought to require experience and time can be efficiently, objectively, and precisely accomplished by AI in the future [39, 40]. By labeling and training on auxiliary examination data, AI can directly examine and diagnose diseases, such as dental caries [18–20], periodontal diseases [12–14], and malocclusions [41, 42], using various sources like CT scans, intraoral scans, facial scans, and photographs. Subsequently, through training on the treatment planning of thousands of cases, AI assists in making treatment decisions, improving the efficiency of junior practitioner in examinations, diagnoses, and treatment planning while minimizing user-related inaccuracies [43, 44]. In the field of dental esthetics, 3D intelligent esthetic measurements efficiently capture abnormal values. Subsequently, AI is employed to directly incorporate these values into prostheses, eliminating the need for complex design procedures. Moreover, interdisciplinary treatment plans, that including orthodontics, orthognathics, and periodontal crown lengthening design, can be visualized at each step through virtual patient [2]. Combined with predictions of soft tissue dynamics during facial expressions, dynamic treatment outcome predictions become possible [24]. This not only assists clinicians in making treatment decisions but also enhances communication with patients, improving doctor-patient interaction. The ability to present different treatment scenarios enables patients to better understand the treatment process and actively participate in decision-making [2].

In the previous study, digital smile design was mainly used for treatment planning and improving doctor-patient communication, with its design outcomes serving as a reference rather than directly used in the final restorations [8, 45]. Base on the results of this study, it was found that 3D esthetic analysis workflow was more accurate in measuring tooth-related parameters than 2D measurements. By utilizing the 3D esthetic analysis workflow established in this study, the measuring value may potentially assist in directly guiding the design and manufacture of prosthesis. This approach is expected to address the problem of the disconnect between 2D measurement and 3D prosthesis design. However, this study serves as a foundational exploration into the application of AI. In subsequent researches, an extensive dataset regarding facial and intraoral scans aims to be collected and thoroughly labeled with the key esthetic points and contours proposed in this study for training the AI recognition system. Following the AI training, applications that utilize the proposed esthetic formula to automatically calculate esthetic parameters are intended to be developed. While our approach shows promise, the primary limitation lies in the resource-intensive nature of AI training. The need for a large dataset and the time-intensive labeling and training processes present challenges.

## Conclusion

In this study, a 3D esthetic analysis workflow of 3D virtual patient was established, demonstrating a high level of consistency and reliability surpassing 2D measurements in the precision of tooth-related parameter analysis. A series of calculation formulas were established for the esthetic analysis and key esthetic points and contours were selected, which serve as crucial elements for AI automation in recognition and enhanced calculation, leading to rapid and accurate esthetic measurements. The workflow, defined as key point detection, coordinate system redetermination, and esthetic parameter calculation, serves as a fundamental study for artificial intelligent esthetic analysis and has laid essential groundwork for the use of artificial intelligence in the field of esthetic dentistry.

## Data Availability

All data generated or analysed during this study are included in this published article.

## Abbreviations

DSD: Digital smile design
AI: Artificial intelligence
3D: Three-dimensional
2D: Two-dimensional
PLY: Polygon file format
OBJ: Wavefront object format
DSLR: Digital single-lens reflex
